# Use of bone callus as a source of bone graft for concurrent tibial malunion repair and contralateral pantarsal arthrodesis in a domestic shorthair cat—a case report

**DOI:** 10.3389/fvets.2025.1665297

**Published:** 2026-01-02

**Authors:** Gracia Tian, Jayson Tuan

**Affiliations:** Department of Surgery, Veterinary Emergency and Specialty Hospital, Singapore, Singapore

**Keywords:** malunion, bone callus, bone graft, pantarsal arthrodesis, feline, dorsal plate, autograft

## Abstract

A domestic shorthair cat, estimated 1 year old, was presented from a local rescue with traumatic injuries of unknown cause to both pelvic limbs. Radiographs revealed a left tibial malunion with marked callus formation and complex right tarsocrural joint injuries. A modified chipping technique was used to provide a source of autogenous bone graft from the callus for both the malunion correction and the contralateral pantarsal arthrodesis. Post-operative radiographs showed significant improvement in the left tibial alignment and adequate arthrodesis of the right tarsal joints. There was good functional outcome at 3 months post-operatively. This case report highlights the potential of bone callus to be used as a bone graft, presenting a unique technique to avoid donor site morbidity associated with traditional autogenous bone grafts. The modified chipping technique can also be employed in malunion revision surgeries to effectively correct resultant angular deformities while preserving the fracture haematoma. To the authors' knowledge these have not been reported in the veterinary literature.

## 1 Introduction

Tibial fractures are common injuries accounting for approximately 10%−20% of all fractures in cats and surgical intervention is often recommended (1). Bone healing without adequate reduction can result in malunions, compromising form and resulting in functional deficits. As a result, corrective surgical osteotomy procedures with bone graft should be considered in such situations (2). A chipping technique has been described in people to address non-union with malalignment following femoral fracture repair surgeries (3). The technique involves revision surgery with chipping of non-union bone without harvesting bone graft from a separate site. The technique was also replicated in a human case report to correct a tibial malunion with resultant angular limb deformity (4), showing promise for the application of such technique in cases of fracture healing complications.

Tarsal arthrodesis can produce good functional outcomes and is a salvage procedure in severe tarsal injuries. The use of cancellous bone graft is one of the basic principles of arthrodesis (5). Autogenous bone graft remains the gold standard and are harvested from known donor sites. However, this carries known risks such as donor site morbidity and contributing to prolonged surgical time and associated complications (6). Potential benefits and use of bone callus in human surgery has been investigated and demonstrate promise for use as bone graft material (7). The histopathological behavior of bone in response to trauma stimulates changes evident in early-stage bone callus (8). In particular, early-stage bone callus has been reported to have increased bone activity and was successfully used to stimulate bone growth when implanted into mice models (7). However, to the authors' knowledge, the use of bone callus as bone graft material in veterinary literature is lacking.

This case report presents the clinical application of a modified chipping technique to harvest bone callus in a domestic shorthair cat for tibial malunion correction and concurrent contralateral pantarsal arthrodesis.

## 2 Patient information

A 2.89 kg, juvenile, female neutered, Domestic Shorthair cat was presented from a local rescue with injuries of unknown cause to both pelvic limbs. Limited history was available as the patient was an urban community animal.

## 3 Clinical findings

On physical examination the patient was bright, alert, and responsive on presentation but was non-ambulatory. There was a palpable angular abnormality of the left pelvic limb, with a perceived tibial procurvatum deformity on visual examination. Reduced range of motion of the right tarsocrural joint was found. No open wounds were observed. No other significant abnormalities were found on physical examination.

## 4 Diagnostic assessments

**Figure 1:**
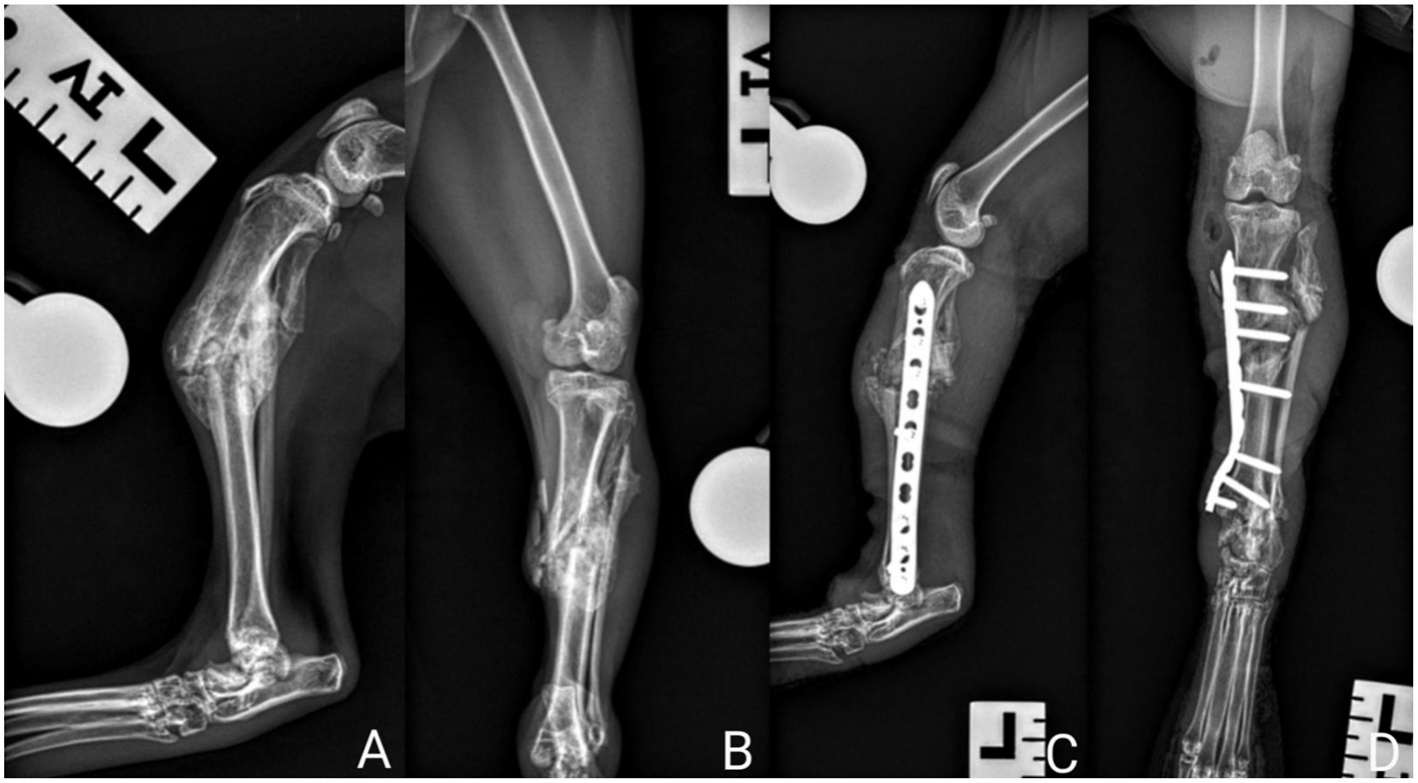
Left hindlimb radiographs. **(A)** Pre-operative mediolateral projection **(B)** Pre-operative craniocaudal projection **(C)** Post-operative mediolateral projection **(D)** Post-operative craniocaudal projection.

**Figure 2:**
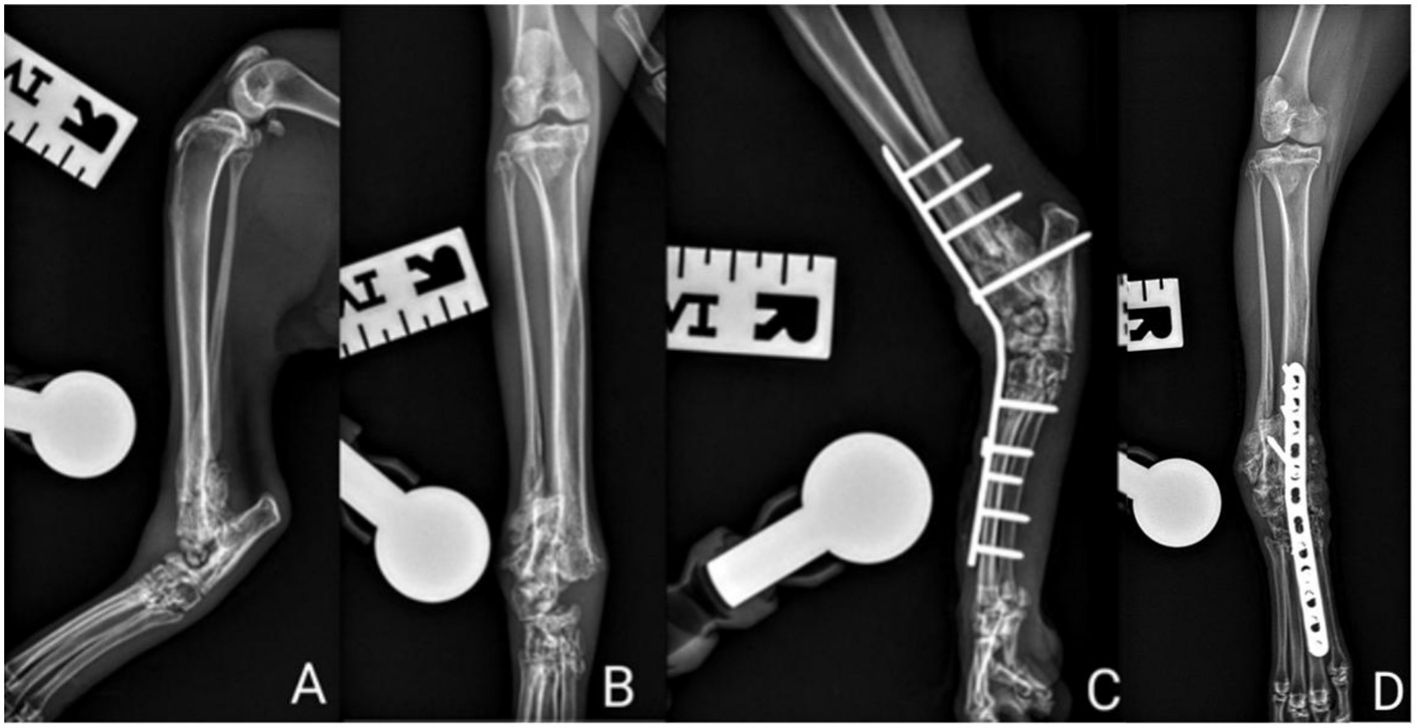
Right hindlimb radiographs. **(A)** Pre-operative mediolateral projection **(B)** Pre-operative craniocaudal projection **(C)** Post-operative mediolateral projection **(D)** Post-operative craniocaudal projection.

Orthogonal radiographs of both pelvic limbs were taken for assessment of injuries (Figures 1, 2). The patient was estimated to be approximately 10–13 months old given the presence of the distal femoral, proximal, and distal tibial fibular growth plates on the radiographs (9).

Mediolateral and craniocaudal radiographs of the left pelvic limb (Figures 1A, B) revealed a complete, malaligned, overriding, comminuted tibial mid-diaphyseal fracture with caudal and lateral displacement. An incomplete bridging bony callus was observed enveloping the fracture site. These changes were consistent with a malunion. In addition, there was a long oblique, displaced fibular fracture with significant overriding with periosteal new bone formation over the lateral aspect of the distal fragment. Mild-moderate soft tissue swelling was observed radiographically over the left tarsocrural joint, with no obvious osseous pathology. On the mediolateral view, a fissure was observed within the talus with evidence of bone remodeling on the dorsal aspect of the bone. Assessment of this injury was not possible on the craniocaudal projection due to the exclusion of the distal limb in the radiograph.

Joint measurements of the left tibia were taken to evaluate the degree of malalignment and assess for adequate degree of surgical correction (Table 1).

**Table 1:** Joint angle measurements pre-operative, post-operative as compared to normal joint angles.

**Joint angles**	**Normal (15)**	**Pre-operative**	**Post-operative**
Tibial plateau angle (TPA)	23.1°± 3.2°	44.6°	22.2°
Mechanical medial proximal tibial angle (mMPTA)	93.5°± 1.2°	95.9°	90.7
Mechanical medial distal tibial angle (mMDTA)	100.5°± 2.3°	93.7°	91.1

There is an improvement in the joint angle measurements obtained post-operatively showing a more normal joint angle has been achieved following surgical correction.

The mediolateral radiograph of the right pelvic limb (Figure 2B) revealed marked bone remodeling at the distal tibial and fibular epiphysis which were not clearly recognizable. The right talus appeared irregular and fragmented. The craniocaudal radiographic projection (Figure 2A) revealed comminuted complex talar and calcaneal fractures with lateral displacement relative to the distal tibial margin. A concurrent subluxation of the proximal intertarsal joint was observed with new bone formation observed lateral to the calcaneus and moderate soft tissue swelling. The epiphysis of the tibia cannot be identified in the distal aspect of the tibia, raising the suspicion of a possible physeal fracture. The distal aspect of the fibula cannot be assessed.

Further diagnostics were not employed due to the patient belonging to a shelter which limited financial resources and access to the patient.

Given the young age of the patient and the presence of an early-stage bone callus, the authors hypothesized that correction of the malunion would likely be successful, and the bone callus harvested during the process could be used as bone graft material.

## 5 Therapeutic intervention

### 5.1 Anesthesia and analgesia

The patient was pre-medicated with a combination of medetomidine (7m cg/kg, Sedator, Dechra, UK), ketamine (2 mg/kg Ketamine, Ceva, Australia) and methadone (0.4 mg/kg, Methone, Ceva) administered intramuscularly. Anesthesia was induced with alfaxalone (titrated to effect, Alfaxan Multidose, Zoetis) and maintained with isoflurane following endotracheal intubation.

Analgesia was provided via fentanyl (Fentanyl-hameln Injection, Germany) as a continuous rate infusion (CRI) with adjunctive diazepam (0.2 mg/kg Pamlin, Ceva, Australia) and ketamine (1 mg/kg, Ketamine, Ceva, Australia) as required to maintain adequate anesthetic depth and analgesia.

Intravenous fluid support with compound sodium lactate solution was given at 3 ml/kg/hour with intermittent boluses of 10 ml/kg/hour as needed to maintain adequate perfusion. Vital parameters were monitored every 5 min by a trained anesthesia nurse.

### 5.2 Surgical procedure

The patient was positioned in dorsal recumbency with both pelvic limbs individually draped into the surgical site using the hanging limb method. A medial approach to the left tibial diaphysis was first performed. Following identification and retraction of the saphenous neurovascular bundle, iatrogenic refracture of the tibial malunion and associated fibula was performed with a combination of oscillating saw, osteotome and mallet. In addition, the bone callus centered over the proximal third of the left tibial diaphysis was carefully fragmented using an osteotome with mallet and a small curved rongeur. Following that, the bone callus fragments were chipped into smaller pieces and kept covered with sterile saline moistened gauze sponges.

The ends of the proximal and distal tibial segment, adjacent to the malunion, was determined via intraoperative identification of the caudal tibial cortex. Transverse osteotomies for each segment were performed using an oscillating saw perpendicular to the caudal tibial cortex. The osteotomies were performed to preserve as much limb length as possible and to provide transverse opposing surfaces following reduction and compression. The excised bone was also fragmented and added to the bone callus chips. Following manual reduction, a 10-hole 2.4 mm Combination Locking Compression Plate (Veterinary Orthopedic Implants, VOI) was contoured and placed on the medial surface of the tibia as a compression plate with hybrid fixation.

Following this, a pantarsal arthrodesis was performed in the right pelvic limb based on previously described technique by Fitzpatrick et al. (10). A cranio-medial approach was made to the right tarsus and a combination of blunt dissection, elevation and retraction of the cranial tibial tendon and muscle was performed. Incision of the joint capsule and hyperextension of the tarsus exposed articular joint surfaces. All articular cartilage was meticulously debrided with a high speed bur.

A second incision was made proximally to expose the distal tibia and an epiperiosteal tunnel was created to connect the incisions. A third incision was made distally at the mid-diaphysis of the third metatarsal bone and the epiperiosteal tunnel was extended. A contoured 12-hole 2.0 mm Combination Locking Compression Plate (Veterinary Orthopedic Implants, VOI) was inserted through the first incision and manipulated to exit the distal incision. The plate was then adjusted to enter the proximal incision and positioned such that the bend of the plate was located at the junction of the talar ridge and neck as the tarsus was held in perceived normal standing angle. Numbering the plate holes 1–12 from proximal to distal, a 2.0 mm cortical screw was placed in hole 9, ensuring accurate placement on the dorsal midline of the third metatarsal bone. This was followed by metatarsal 2.0 mm locking screws in holes 8 and 12. With the tarsus held in normal alignment and the bend of the plate in opposition to the talus, the screws in holes 2 and 3 (2.0 mm locking) were placed on the cranial midline of the tibia. The remaining tibial screws in hole 1 and metatarsal holes 10 and 11 were subsequently placed (2.0 mm locking). Lastly, a tibiocalcaneal screw (2.0 mm cortical) was then placed through plate hole 5. The previously placed tibial locking screws were slightly loosened prior to complete tightening of this tibiocalcaneal cortical screw. Following this, these tibial locking screws were retightened.

Both surgical sites were lavaged with sterile saline and the tibial malunion site and the tarsal joint spaces were loosely packed with the harvested bone callus fragment chips. All incisions were closed routinely.

### 5.3 Post-operative care

The patient recovered from anesthesia uneventfully. A modified Robert Jones bandage was placed on the right pelvic limb to provide temporary mechanical support to the pantarsal arthrodesis during the healing period—this was removed in 1 week. The patient returned to the shelter for post-operative management with instructions for cage rest of 8 weeks duration before a gradual introduction in increased activity. Meloxicam 0.05 mg/kg PO q 24 h was dispensed for 7 days for analgesia and the patient was to wear a buster collar for 2 weeks to prevent any self-trauma.

Adequate wound healing was reassessed by the shelter staff and no further intervention was required.

## 6 Follow-up and outcomes

### 6.1 Post-operative radiographs

Immediate post-operative radiographs performed (Figures 1C, D, 2C, D) confirmed adequate reduction, alignment, and implant positioning of the left tibia and right tarsocrural joint.

Joint measurements were performed with the pre- and post-operative radiographs. An improvement in tibial plateau angle was achieved post-operatively (Table 1).

The inclusion of the distal left hindlimb in the craniocaudal projection allowed for evaluation of the tarsocrural joint. There was additional bone formation lateral to the talus on the craniocaudal projection. On the mediolateral projection there was dorsal displacement of the dorsal talar fragment. These changes did not appear to have a clinical effect and further surgery was not pursued.

### 6.2 Clinical reassessment

A recheck examination was not performed as the patient returned to the shelter and was subsequently under the care of a fosterer. Follow up videos provided demonstrated adequate function post-operatively where the patient can be seen running, walking, jumping and fully weight bearing with no overt lameness. There were no reports of concerns with the surgical sites up to the point of final follow up.

On pictures and videos provided, no obvious difference in limb length can be observed. Despite a mild perceived external rotation of the left hindlimb, this did not appear clinically significant as the patient was observed to be fully weight bearing on both hindlimbs.

### 6.3 Follow up imaging

**Figure 3:**
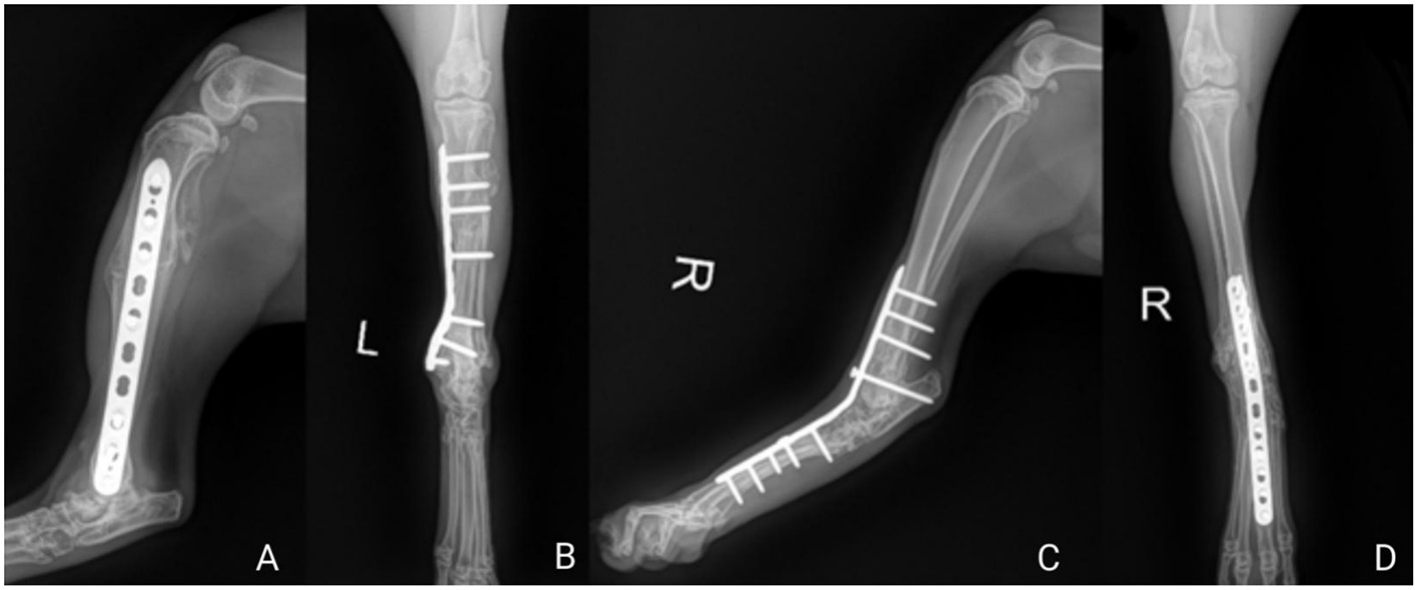
Two month post-operative radiographs of the left and right hindlimbs showing successful union and fusion. **(A)** Left hindlimb mediolateral projection. **(B)** Left hindlimb craniocaudal projection. **(C)** Right hindlimb mediolateral projection. **(D)** Right himdlimb craniocaudal projection.

Radiographs were performed at the shelter at 1 and 2 months (Figure 3) post-operatively to assess fracture healing and arthrodesis.

Bone remodeling with successful union of the left tibial fracture can be appreciated. Union and remodeling of the cranial fibular fragments can be observed on both lateral and cranial-caudal projections with evidence of synostosis at the previous fracture site. Evidence of bone remodeling was also present within the left talus in both projections. Based on the reported clinical function of the cat, the tarsal changes did not appear to be clinically significant.

At the final radiographic assessment, adequate arthrodesis was achieved in the right tarsus with presence of bony bridging across the tarso-crural, intertarsal, and tarsometatarsal joints. However, the subchondral bone plates remained partial visible in the intertarsal and tarsometatarsal joints.

## 7 Discussion

Complex fractures of multiple limbs are clinically challenging and can require advanced surgical techniques to provide a functional outcome for the veterinary patient. In community animals, such injuries in a single limb would often result in amputation due to financial limitations of rescue work and the perceived acceptable quality of life (11). However, in situations with multiple limbs involvement, limb preservation should be pursued.

The tibial malunion and resultant angular limb deformity in this patient resulted in a non-functional limb. This is further compounded by a contralateral pelvic limb injury. Corrective procedures involving osteotomies and ostectomies can be performed, but the high rate of complications for feline tibial fracture repair needs to be taken into consideration. Suggested factors include limited blood supply, an excessive fracture gap and inadequate stabilization (12). A closing wedge osteotomy and stabilization with external skeletal fixator have been reported to correct a similar tibial malunion in a young dog (2). However, external fixation was associated with higher complication rates in feline patients when compared to open reduction and internal fixation (13). As a result, internal fixation was the preferred method of stabilization in this patient following correction of the malunion using a modified chipping technique.

The presence of a concurrent complex right tarsal injury further increased the clinical challenge in this case. Pantarsal arthrodesis is a well-established salvage procedure in veterinary literature and is indicated in a range of tarsal injuries, providing acceptable long-term function (10). The basic principles of surgical arthrodesis include meticulous and complete removal of articular cartilage at intended fusion sites, apposition of joint surfaces at a functional standing angle, robust internal fixation using compression, use of a cancellous bone graft, and careful preservation of soft tissue (5). Autogenous bone graft from a separate donor site or commercially available allograft products are options for bone graft (14), with autogenous bone grafts being superior due to histocompatibility and osteogenic properties. However, autogenous bone graft requires an additional procedure on a separate donor site, adding to surgical time and increasing the risk of complications.

A “chipping” technique has been reported to be successful in the correction of a femoral non-union with malalignment. The technique was hypothesized to improve fracture healing due to introduction of mesenchymal cells and/or progenitor cells and inductive molecules from bone marrow to the fracture site (3). The technique was also reported to have produced successful outcomes in a case of tibial malunion with angular deformity in human literature (4). The successful outcomes reported in the human literature showed promise to the application of the technique when addressing certain fracture healing complications. To the authors' knowledge, this technique has yet to be reported in veterinary literature.

A study in human literature reported on the osteogenic properties of early-stage callus (defined as callus less than 3 months) and therefore it's potential for use as autograft material (7). The physiological responses of bone to the fracture micro-environment results in an increased amount of bone activity within the early fracture callus (7, 8) making it an osteogenic and osteoinductive material. The ability of early-stage bone callus to induce new bone formation was reported following implantation into a nude mice model (7). As such, the bone callus chips produced from this reported “chipping” technique was theorized to encompass these properties and were therefore utilized as bone graft material in both surgical sites in the patient in this case report. Follow up radiographs performed showed evidence of adequate bone union in the revised alignment with improvements in the TPA in the left hindlimb. The pantarsal arthrodesis performed in the right hindlimb was also noted to have adequate joint fusion. These findings strongly support the potential clinical application of the modified “chipping” technique and the purported benefits of bone callus as autograft material.

One of the biggest limitations of this being a case report would be the sample size. Although a successful outcome was achieved, we are unable to conclude that successful outcomes can be obtained reliably. Potential complications could include a non-union of the fracture site following the chipping technique or lack of adequate osteogenesis in the pan-tarsal arthrodesis site. However, these complications could potentially be addressed in a revision surgery. More cases would be required to further support the use of this technique and the use of bone callus as a autograft material. Case reports are also limited by the lack of consistency, especially with the patient reported being a shelter animal. While the procedure was a single-stage surgery performed by a single surgeon, there was a lack in consistency with personnel performing the pre- and post-operative radiographs or treatments. Advanced imaging such as computed tomography (CT) could have been utilized for more consistency but financial limitations did not allow this. The animal was also subsequently returned to the shelter then fostered and subsequently adopted, making follow up difficult.

Another limitation of the case report is the unknown stage of the bone callus. The incomplete appearance of the callus on the radiographs performed, the presence of soft tissue swelling surrounding the fracture site and the patient's young age support the hypothesis that the injury was recent and bone callus formed would be of early stage. However, no histopathological analysis of the bone callus was performed. The differences between early vs. late-stage callus resulted in varied osteogenic ability in the human study (7) which could affect the outcome when callus of different stages are used as an autograft. However, the case of the tibial malunion correction using chipping osteotomy achieved good outcomes despite a prolonged period from time of injury to corrective surgery (4) which could suggest that early-stage callus is not necessary in use of the chipping technique in clinical application. The successful osteogenesis in the right pantarsal arthrodesis supports the assumption of the osteogenic ability of the callus material harvested in the patient but histopathological analyses would be needed to confirm exact staging of the bone callus.

## 8 Conclusion

The use of a modified “chipping” technique in a cat was successful and produced a functional outcome. Bone callus from fracture sites may be used as an alternative to autogenous bone graft material to yield similar benefits while negating the risks involved in harvesting from a separate donor site. More cases reporting on the use of these techniques will be needed to further support their use in veterinary literature and reliability of the outcomes.

## Data Availability

The original contributions presented in the study are included in the article/Supplementary material, further inquiries can be directed to the corresponding author.

## References

[1] Hayashi K Kapatkin AS. Fractures of the tibia and fibula. In:Johnston SA Tobias KM, editors. Veterinary Surgery: Small Animal. Missouri: Elsevier (2018). p. 1176–91.

[2] Kraus KH Bayer BJ. Delayed unions, nonunions, and malunions. In:Johnston SA Tobias KM, editors. Veterinary Surgery: Small Animal. Missouri: Elsevier (2018). p. 172–761.

[3] Watanabe T Matsushita T. Femoral non-union with malalignment: reconstruction and biological stimulation with the chipping technique. Injury. (2016) 47:S47–52. doi: 10.1016/S0020-1383(16)30839-7 28040087

[4] Yusof N. Tibia malunion with angular deformity: corrective osteotomy and intramedullary fixation with the chipping technique. Biomed J Sci Tech Res. (2018) 2:000628. doi: 10.26717/BJSTR.2017.01.000628

[5] Carmichael S Marshall WG. Tarsus and metatarsus. In:Johnston SA Tobias KM, editors. Veterinary Surgery: Small Animal. Missouri: Elsevier (2018). p. 1193–219.

[6] Cheng H Clymer JW Chen BPH Sadeghirad B Ferko NC Cameron CG . Prolonged operative duration is associated with complications: a systematic review and meta-analysis. J Surg Res. (2018) 229:134–44. doi: 10.1016/j.jss.2018.03.022 29936980

[7] Han W Gao C Liu J Yue H Yu Y Wang X . The osteogenic potential of human bone callus. Sci Rep. (2016) 6:36330. doi: 10.1038/srep36330 27796345 PMC5087090

[8] Papachristou DK Georgopoulos S Giannoudis PV Panagiotopoulos E. Insights into the cellular and molecular mechanisms that govern the fracture healing process: a narrative review. J Clin Med. (2021) 10:3554. doi: 10.3390/jcm10163554 34441849 PMC8397080

[9] Miranda FG Gouveia CH Ferreira RF Vieira LG Borges NC. Radiographic study of development of pelvis and hip and femorotibial joints in domestic cats. J Feline Med Surg. (2020) 22:476–83. doi: 10.1177/1098612X19854809 31184248 PMC10814335

[10] Fitzpatrick N Sajik D Farrell M. Feline pantarsal arthrodesis using pre-contoured dorsal plates applied according to the principles of percutaneous plate arthrodesis. Vet Comp Orthop Traumatol. (2013) 26:399–407. doi: 10.3415/VCOT-12-05-0063 23612609

[11] Forster LM Taylor SM Shmon CL Bersenas AME Wuerz TH. Owners' observations of domestic cats after limb amputation. Vet Rec. (2010) 167:734–9. doi: 10.1136/vr.c5893 21257508

[12] Zurita M Craig A. Feline diaphyseal fractures: management and treatment options. J Feline Med Surg. (2022) 24:662–74. doi: 10.1177/1098612X221106354 35775308 PMC11107983

[13] Perry KL Bruce M. Impact of fixation method on postoperative complication rates following surgical stabilization of diaphyseal tibial fractures in cats. Vet Comp Orthop Traumatol. (2015) 28:109–15. doi: 10.3415/VCOT-14-08-0120 25650891

[14] Harasen G. Stimulating bone growth in the small animal patient: grafts and beyond! *Can Vet J*. (2011) 52:199–200. PMC3022466 21532832

[15] Palumbo Piccionello A Di Pietro S D'Angelo A Macrì F Della Valle G Cassata G . Good inter- and intra-observer reliability for assessment of radiographic femoral and tibial frontal and sagittal planes joints angles in normal cats. Vet Comp Orthop Traumatol. (2020) 33:308–15. doi: 10.1055/s-0040-1709694 32408358

