# Supplementary figures and images for: Use of bone callus as a source of bone graft for concurrent tibial malunion repair and contralateral pantarsal arthrodesis in a domestic shorthair cat—a case report
Source: Front Vet Sci. 2026 Jan 2;12:1665297. doi: 10.3389/fvets.2025.1665297 (PMC12807949; doi:10.3389/fvets.2025.1665297)

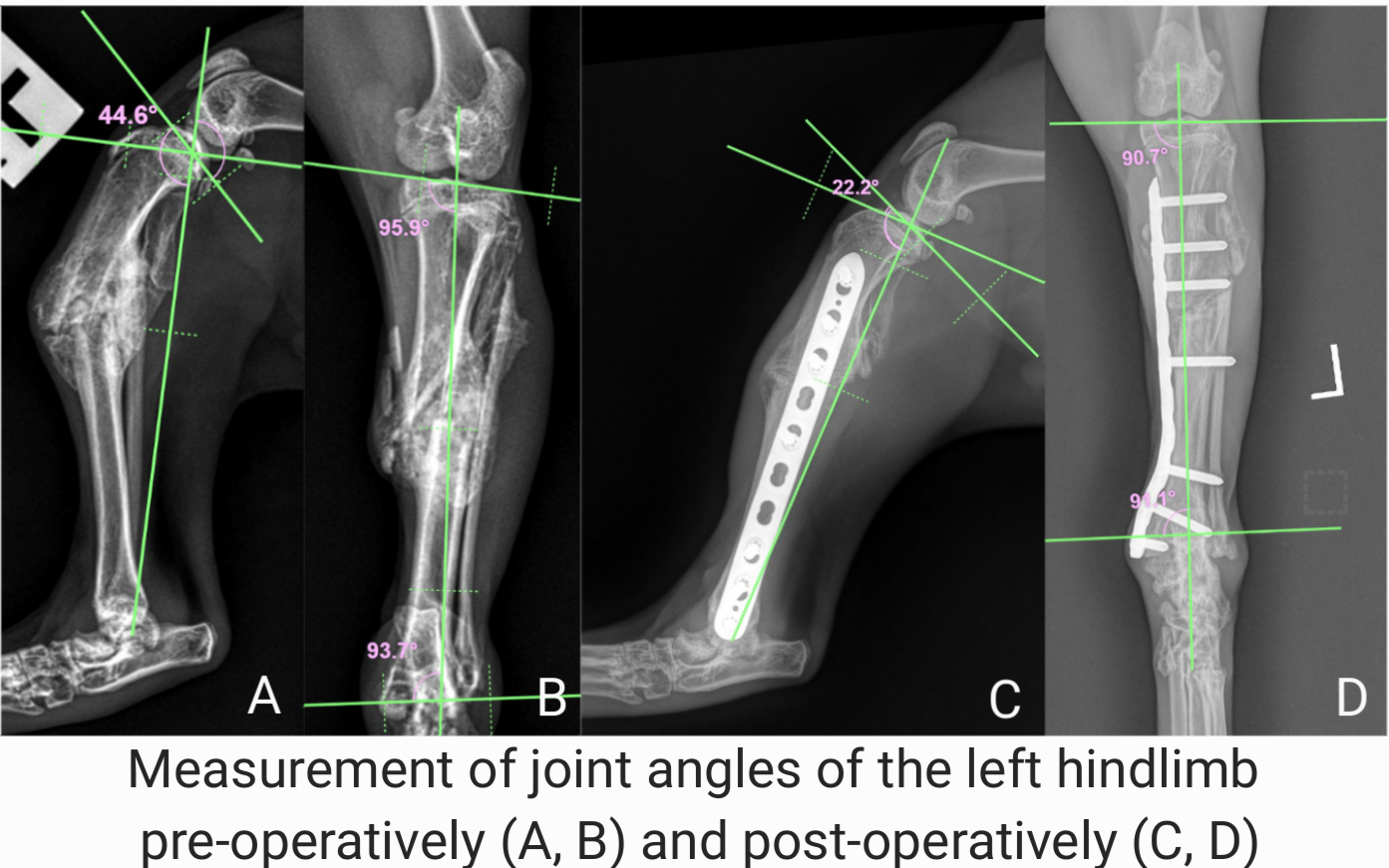

Supplement: Supplementary file 1 [file Image_1.png]

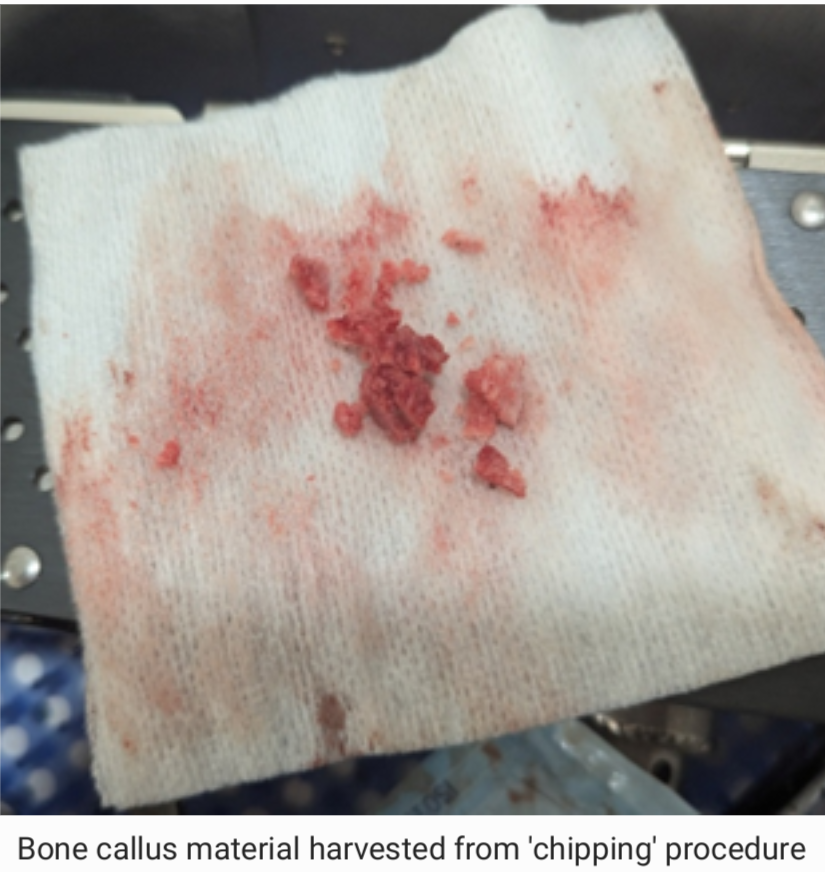

Supplement: Supplementary file 2 [file Image_2.png]

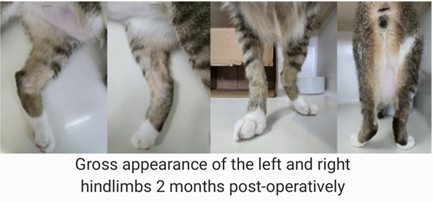

Supplement: Supplementary file 3 [file Image_3.png]

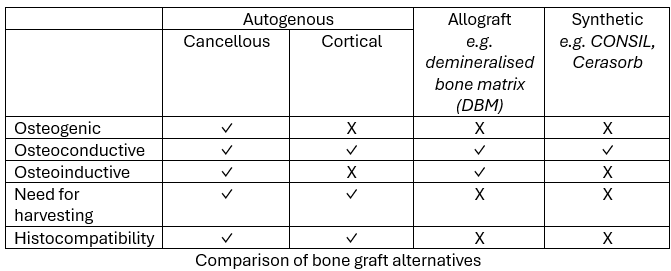

Supplement: Supplementary file 4 [file Image_4.png]
